# Editorial: Nematodes: an integrated pest management approach

**DOI:** 10.3389/fpls.2023.1355670

**Published:** 2024-01-10

**Authors:** Mahfouz M.M. Abd-Elgawad, Francesca De Luca, Tarique Hassan Askary

**Affiliations:** ^1^ Plant Pathology Department, National Research Centre, Giza, Egypt; ^2^ Istituto per la Protezione Sostenibile delle Piante, Consiglio Nazionale delle Ricerche, Bari, Italy; ^3^ Division of Entomology, Faculty of Agriculture, Sher-e-Kashmir University of Agricultural Sciences and Technology of Kashmir, Sopore, Jammu and Kashmir, India

**Keywords:** biocontrol, crop yield, molecular and non-molecular interactions, plant-parasitic nematode management, resistance

Plant-parasitic nematodes (PPNs) cause severe crop damage and yield losses on economically important crops worldwide. A recent report of such losses for the 40 life-sustaining crops with impressing figures for food and export recorded an average of 13.5% retardations; equalled USD 358.24 billion annually (Abd-Elgawad, 2023). Traditional nematicides, despite effective in nematode control, have been banned because of concerns for the environment and human health, the aim of this Research Topic is to highlight new research on integrated pest management (IPM) of PPNs exploring innovative approaches for sustainable and effective control of PPNs. These strategies involve research on biocontrol agents, cultural methods, plant resistance and functional genomics in order to achieve safer and effective control of PPNs (Abd-Elgawad, 2022).

Gohar et al. focused on the use of two quantitative approaches, assignment Cant-Saenz’s (AQSCS) and modified host-parasite index (MHPI), and single nucleotide polymorphisms (SNPs) as molecular markers to distinguish among different sugar beet genotypes as tolerant, susceptible or resistant towards the root-knot nematode (RKN) *Meloidogyne incognita.* Meanwhile, the multiple parameters-based rating revealed significant differences among beet genotypes concerning yield production, disease severity, and quality traits. Finally, the screened high-yield genotypes with tolerance/resistance to RKNs can help sugar beet breeders to generate novel commercially desirable genotypes. In addition, SNPs markers can help to efficiently identify resistant and susceptible genotypes.

Saikai et al. study evaluated the efficacy and safety by using two biocontrol agents (BCAs), *Trichoderma asperellum* and *Purpureocillium lilacinum*, for *Meloidogyne hapla* control in coffee fields. Such BCAs have merits over chemical pesticides mostly as safe methods that can suppress RKN pests under economic threshold levels for sustainable agricultural programmes (**Figure 1**). The application of both biocontrol agents produced a reduction of RKN densities and consequently less damage to coffee trees. Furthermore, the evaluation of the impact on the soil nematode community revealed that *T. asperellum* improved soil health conditions via increasing the diversity of the microbial community, while *P. lilacinum* increased the presence of fungivorous nematodes. This study showed the potential of drenching biocontrol agents in coffee fields in Kenia for controlling *M. hapla* and their positive impact on soil health.

**Figure 1:**
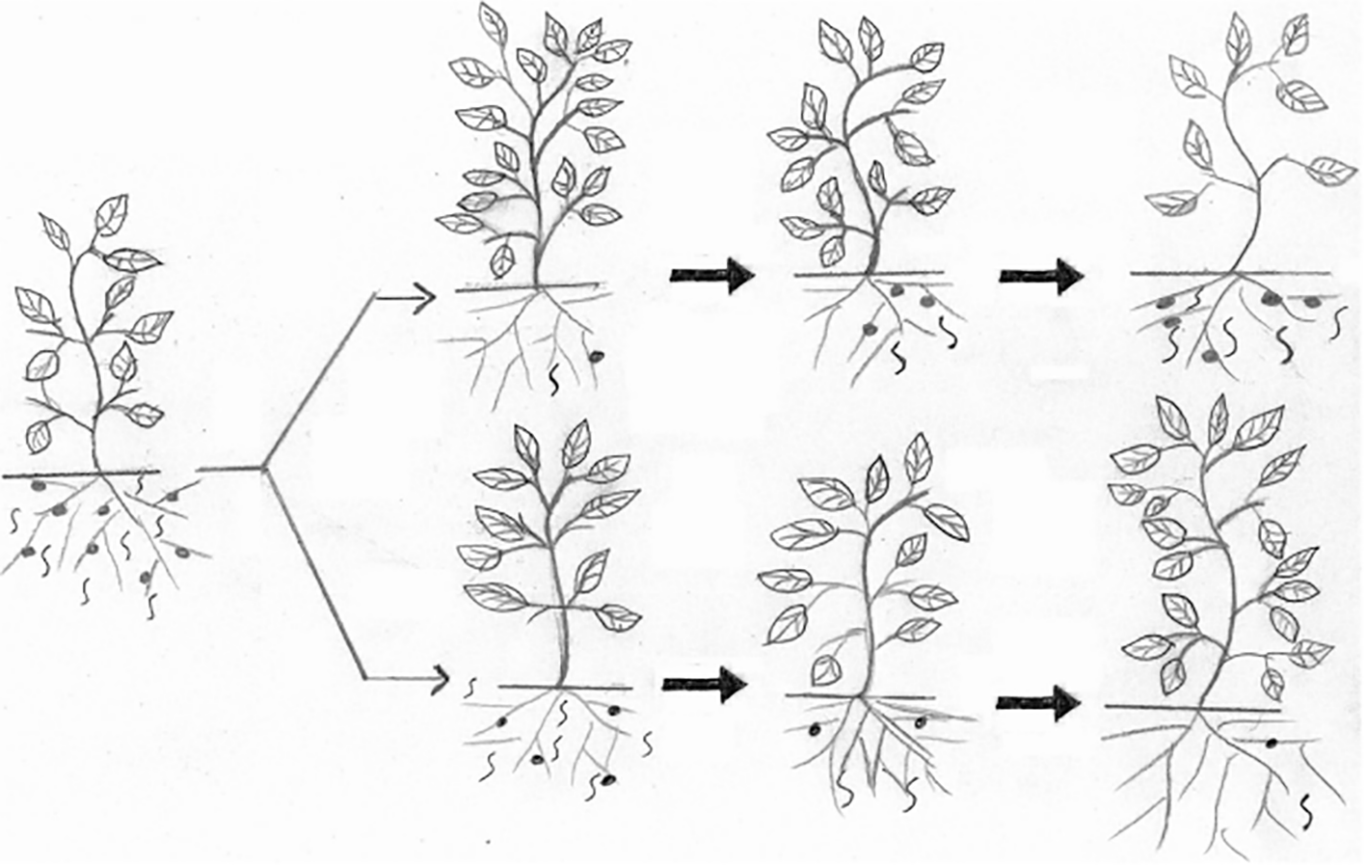
Effect of a chemical nematicide (upper) and a bionematicide (lower) trend on root-knot nematodes on susceptible plants. On applying both nematicides, the chemical has rapid and significant effect on reducing the nematode population, but a few nematodes can escape its effect and reproduce to reach damaging level while the bionematicide can work continuously to keep the nematode below the economic threshold level (Abd-Elgawad and Askary, 2020).

The study of Cardoso et al. focused on the characterization of a large set of differentially expressed genes (DEGs) in the transcriptome of *Meloidogyne luci* infective second stage juveniles in response to 1,4-naphthoquinone treatment (1,4-NTQ) using RNA-seq approach. In a previous paper, Maleita et al. (2022) demonstrated that the 1,4-NTQ had nematicidal activity on *M. luci* reproduction and little is known about the way of acting of this phytochemical. Thus, the analyses of DEGs in response to 1,4-NTQ treatment revealed a high number of downregulated genes compared to the control, most of them related to ribosomes and thus to translation mechanism, were affected by 1,4-NTQ. This result contributed to understand how this bionematicidal compound interacts with nematodes. Furthermore, this finding will open a novel avenue for its sound/effective use among next generation nematicides.

Sood et al. study focused on genome-wide association studies (GWAS) and genomic prediction (GP) for potato resistance to three serious pathogens *Phytophthora infestans*, *Globodera pallida*, and *G*. *rostochiensis. Globodera pallida* and *G*. *rostochiensis* can inflict major damage to potato production due to their virulence, spread, and adaptability. Genotyping and SNP marker analyses revealed the suitability of specific single nucleotide polymorphisms (SNPs) from the selection of potato accessions resistant to the potato cyst nematodes (PCNs). A total of 7 SNP associations for *P. infestans* resistance, 9 and 11 for *G*. *pallida* and *G*. *rostochiensis*, respectively, were uncovered by additive and simplex dominance styles of GWAS. These results offer useful SNP markers to ease breeding for potato resistance for both the PCNs and the pathogenic fungus.

Overall, the papers published within the Research Topic, *Nematodes: An Integrated Pest Management Approach*, are so useful and attractive. The multiple parameters-based rating for host suitability designation of nematode resistance/susceptibility incorporates molecular tools for such a superior designation to develop improved varieties. Using the BCAs are effective measures that are undoubtedly to be taken for their safety in controlling nematode pests while boosting soil health. Grasping the mechanism of bio-nematicidal compound such as 1,4-NTQ in controlling nematodes may pave the way for reliable and novel nematicidal generation. Molecular approaches of SNP markers to expedite potato breeding programs for nematode and fungal resistance are advanced genetic breeding techniques that can complement classical breeding with the sophisticated genetic methods. In summary, the aforementioned studies can contribute in laying the significant groundwork and showcasing promising progress in identifying upcoming and new technologies for integrated pest management for prosperous and sustainable agriculture.

## Author contributions

MA-E: Writing – review & editing, Conceptualization, Data curation. FDL: Conceptualization, Methodology, Writing – review & editing. TA: Conceptualization, Data curation, Investigation, Validation, Writing – review & editing.
